# Change of organizational newcomers’ unmet expectations: Does proactive coping matter?

**DOI:** 10.1371/journal.pone.0243234

**Published:** 2020-12-10

**Authors:** Katarzyna Ślebarska, Roman Soucek

**Affiliations:** 1 Institute of Psychology, University of Silesia in Katowice, Katowice, Poland; 2 School of Business, Economics, and Society, Friedrich-Alexander University Erlangen-Nürnberg, Nuremberg, Germany; Jacobs University Bremen, GERMANY

## Abstract

Unmet expectations are one of the major sources of strain for organizational newcomers. We explore the change of newcomers’ expectations over time and propose that proactive coping should restrict the amount of unmet expectations. We recruited participants among employees from newly opened retail stores (*N* = 172) and accompanied them for six months after organizational entry. The results revealed a change of unmet expectations over time. Though proactive coping was related to a lower amount of unmet expectations right after organizational entry, unmet expectations increased after six months, especially in case of high proactive coping.

## Introduction

The early period of employment is considered as one of the most critical phases of organizational life. Organizational newcomers enter new jobs with some expectations about their future workplace and their role in the organization [1], and the fulfillment of their expectations is an important indicator of a successful organizational socialization [2]. Previous studies have shown the negative influence of unmet expectations on several outcomes, like job satisfaction or turnover intention [1, 3]. The failure to meet new employees’ expectations can even exacerbate the “reality shock” and lead to unsuccessful adaptation and a lower job satisfaction [4]. Regarding these negative consequences, the problem of unmet expectations remains a crucial topic for organizational socialization.

Unmet expectations are defined as the perceived difference between prior expectations and actual experiences in the workplace. Since the actual experience differs in the process of onboarding, the fulfillment of expectations might also change during organizational entry. Contrary to prior conceptions of organizational newcomers as being passive and reactive respondents to their work context, the prevalent view portrays employees as actively shaping their jobs and work environment [5, 6]. This active work behavior refers to proactive coping that describes individuals as self-started, change-oriented, and future-focused [7]. Proactive coping is defined as a specific form of problem-focused coping that involves taking action to prevent stressful events or to cope successfully with future stressors [8]. Proactive coping helps to avoid the occurrence of a stressful situation or limits its influence on a person by strengthening the individual’s capabilities for managing or even changing the situation in advance [8, 9], and therefore, supports the fulfillment of expectations. Proactive individuals initiate a constructive course of action and create opportunities for self-growth by themselves. Thus, proactive employees seek to change their work environment so that it meets their pre-entry expectations.

Overall, this study contributes to our understanding of organizational newcomers’ expectations fulfillment in two ways. First, we perceive the fulfillment of expectations as a process, and therefore highlight the change in expectations over time after organizational entry. Second, we describe how the change in expectations depends on proactive coping.

### Change in unmet expectations

Current approaches to organizational socialization assume that organizational newcomers formulate their expectations before entering a new workplace, usually during the process of recruitment and selection [1]. This early period of organizational entry is followed by a significant amount of uncertainty and stress. Existing research identified unmet expectations as one of the entry stressors that is particularly important for organizational newcomers [10]. Since individuals enter the organization with an idealistic view on working conditions, their expectations often are inconsistent with reality [1]. They mostly anticipate their work as challenging, exciting, and meaningful. If the new job fails to fulfill these presumptions, organizational entrants may feel a tension between their daily work experience and their own professional expectations [11]. Higher discrepancy between initial expectations and encountered conditions may arise from unsatisfying work experiences that result in higher job stress [12]. Although the literature on workplace expectations underlines the importance of an early measurement of expectations [3], the discrepancy between expected and encountered job conditions should be assessed after newcomers have become full members of the organization [13]. Since individuals’ assessment of pre-entry expectations are filtered by more recent experiences and behaviors, it is important to understand the fulfillment of expectations as a process that should be assessed at the very first stage in the new workplace as well as at later stages after organizational entry.

At the first stage of the onboarding process, organizational entrants could experience a kind of honeymoon effect. Even though new employees might feel anxiety or stress, they tend to interpret their situation more positively, and actual differences to their expectations could be perceived as exciting or interesting [14]. Because of various socialization tactics in the entry phase, like orientation and training programs, organizational newcomers are more isolated from the “real” job environment, which could also contribute to the honeymoon effect. For example, mentoring aims at helping new employees to acclimate more quickly and the mentor provides advice in integration issues.

However, while becoming more involved into the new job, formal socialization programs end, and organizational entrants become more exposed to actual job demands and negative experiences like disappointment, frustration, and tension. Additionally, job content plateau and job tenure might cause a lack of enthusiasm and lead to a stronger call for positive change [15]. Since new employees gain new job knowledge and skills at early stages of organizational entrance, a continued development of their tasks and job roles becomes more and more difficult, they reach a job content plateau [16]. Hence, employees’ expectations about the new work environment may change compared to the first phase of organizational entry. The reexamination of differences between initial expectations and encountered work conditions could thus result in the perception of being less fulfilled compared to the time of organizational entry. Although the experience of being hired might result in a more optimistic view and self-confidence in the early days of employment, some evidence indicates that highly optimistic expectations may fade out as a person repeatedly encounters situations that subvert those expectations [17]. With hindsight, the differences between initial expectations and encountered work conditions could thus turn into unmet expectations. We therefore assume that the perceived fulfillment of expectations changes over time. More precisely, we hypothesize that the level of unmet expectations increases after organizational entry (cf. Hypothesis 1).

**Hypothesis 1:** The level of unmet expectations increases over time.

### Unmet expectations and proactive coping

The early period of organizational entry is characterized by uncertainty and unmet expectations are one of the major sources of strain for organizational newcomers [2, 10]. Since individuals often enter organizations with an idealistic view on working conditions, their expectations are often inconsistent with reality. They mostly anticipate their work as challenging, exciting, and meaningful. Expectations are probably based on insufficient information about the actual workplace, either because the newcomers had not gathered enough information or because the organization did not provide information about the workplace. From the organizational perspective, the concept of realistic job previews [13] aims to provide an accurate perception of the new workplace and especially lowers employees’ expectations, which are more likely to be satisfied compared to higher initial expectations [18]. However, from the personal perspective, individuals react differently to similar circumstances, and thus, there is a need to consider both, personal and contextual factors in understanding the perception of the situation and work-related behavior [19]. For example, as newcomers enter an organization they are introduced to other employees’ classifications of working conditions. Positive or negative classifications help them to organize their experiences, to assess the differences between prior expectations, and to encounter working conditions, which gives them possible tools at hand for regulating the situation.

One of the techniques used to regulate and influence one’s own perception of the working conditions is proactive coping. Proactive coping involves taking action to prevent stressful events [8] or to better cope with future stressors [20] by accumulating resources and trying to gain and maintain control of someone’s environment [21]. Since this way of coping helps to avoid a stressor or to decrease its influence on a person [8, 9], it helps to deal with both already existent demands [9] and potential future stressors [8].

In a new work environment, organizational newcomers rather need to take the initiative by preparing themselves in advance for unexpected stressors [22]. To put it in another way, organizational newcomers should engage in proactive behavior [23], particularly proactive coping [21]. Proactive coping includes an anticipation of the situation in the new workplace, which might lead to a more realistic view on the stressful situation of entering a new organization, and therefore, might lead to lower expectations. Furthermore, the anticipation of possible problems at the new workplace includes the generation of strategies to cope with this situation. The newcomers may be particularly proficient at behaving proactively to meet their own job expectations and to adjust positively to the new workplace. New employees who engage in proactive coping, acquire information and gather resources, and therefore, might enter the new workplace with a set of more realistic job previews. Moreover, employees who act proactively, use their experience as well as available resources to get their needs met [24]. Thus, the experience of actual problems at work might be not that severe and prevent expectations from becoming unmet.

Furthermore, since proactive copers have anticipated different scenarios and have accumulated personal and social resources in advance, they should have more realistic expectations and are more broadly prepared to cope with the actual situation in the new workplace, and therefore, should be more likely to succeed in expectations fulfillment. Thus, we assume that proactive coping decreases the amount of unmet expectations (cf. Hypothesis 2).

**Hypothesis 2:** Proactive coping is negatively related to unmet expectations.

Some studies found that newcomers do not only gather information about their tasks and work environment but may effectively modify the working context to make it more suitable to their needs and preferences [25]. Proactive coping is thus described through information and feedback seeking as well as through modifying and positive framing of one’s work environment. Drawing from the typology of proactive coping strategies, proactively behaving employees try to modify their own tasks and attempt to keep a positive outlook. However, positive expectations may sometimes lead them to hold unrealistic goals, and consequently, result in unmet expectations in the long run (cf. Hypothesis 1). Nevertheless, highly positive expectations may not be detrimental for individual well-being if a person possesses the necessary resources and skills for confirming those expectations. Accordingly, recent studies [26] underlined the importance of newcomers’ proactive behaviors for well-being. Newcomers who behave proactively are more likely to address anxiety or uncertainty in their setting, and therefore maintain well-being.

To sum up, if the new job fails to fulfill presumptions, organizational entrants are confronted with an incongruence between their daily work experience and their own professional expectations [11]. A higher discrepancy between initial expectations and actually encountered conditions may arise from unsatisfying work experiences that result in higher job stress [12]. As outlined before, proactive coping helps to deal with both already existing demands as well as potential future difficulties [8, 9]. Therefore, we expect a different development of unmet expectations depending on the level of proactive coping. We hypothesize that proactive coping might ameliorate the “reality shock” after organizational entrance and restricts the increase in unmet expectations (cf. Hypothesis 3).

**Hypothesis 3:** Proactive coping moderates the development of unmet expectations over time: The increase in unmet expectations is lower in case of high proactive coping.

## Materials and methods

### Participants and procedure

The participants of this study were organizational newcomers of electronic retail stores in Poland. The participants were recruited among new employees from three newly opened stores (*N* = 172). Fifty-three per cent of the participants were female and the mean age was *M* = 31.47 years (*SD* = 9.79). Forty-four percent of the participants were previously unemployed. The mean period of unemployment of the previously unemployed participants was *M* = 7.77 months (*SD* = 6.16). The study was designed as a longitudinal study with three waves. The assessments took place at the time of entry into the organization (*N* = 172), three months later (*N* = 119), and six months after organizational entry (*N* = 99). During the three time points of data collection participants received identical questionnaires.

Completion of questionnaires was done on a voluntary basis; no monetary compensation was provided. Participants were informed about the anonymity of data collection, that is, their data would be analyzed collectively, and no information would be provided to third parties. They were assured that there are no wrong answers and that all their opinions are important. Respondents were also informed about the purpose of the study, i.e., coping in the situation of reemployment. Since the study was fully anonymous and the data was gathered only among volunteers, the need for written consent from the participants was waived. To ensure the anonymity of the participants no person-related data were collected. Participants were invited to record an anonymous ID (e.g., nickname) so that individual responses could be matched over time. Prior to participation, they were informed about the possibility to withdraw from the study at any time and provided an oral consent to participate in the study. The current study received approval from the Ethics Committee of the Faculty of Pedagogy and Psychology, University of Silesia in Katowice (decision No. 21/2019). The data for this study was gathered within a project as described in a paper published in the Journal of Career Development [21].

### Measures

Unmet expectations were assessed with 11 items based on previous questionnaires [27], asking for different job conditions (e.g., co-workers, supervisor, duties, payment etc.). All items required participants to rate the extent to which their initial expectations are fulfilled using a 3-point scale from -1 (*less than expected*), 0 (*as expected*) to +1 (*more than expected*). The item scores were reverse coded and averaged; higher scores reflected a lower fulfillment of expectations (labelled as unmet expectations).

Proactive coping was assessed with 14 items of the “proactive coping scale” from the Proactive Coping Inventory; Polish version [28]. A sample item is “When I experience a problem, I take the initiative in resolving it.” The participants answered each item on a 4-point rating scale ranging from 1 (*never)* to 4 (*always*). Higher scores indicate a higher level of proactive coping. Cronbach’s α was α = .88. In order to test for measurement invariance between waves, we computed confirmatory factor analyses separately for the three waves. The results and fit indices were comparable across all waves, indicating no measurement variances.

As control variables, we assessed age, gender, and pre-entry experience. According to previous findings, reactions to similar circumstances can differ among individuals [29], and therefore, we consider in this study an interactionist perspective between personal and situational factors. Since newcomers enter a new organization with a set of experienced stress symptoms, which might be a possible explanation for the individual differences in reactions to stressors [30], we assume that pre-entry status (i.e., previous unemployment) would differentiate employees in the level of expectations fulfillment. Therefore, we expect different development of unmet expectancies for two different groups, depending on their pre-entry experience. In particular, we are going to distinguish between previously unemployed and job changers, i.e. previously employed. The participants were asked whether they were unemployed before the current job; we dummy-coded pre-entry experience with 0 (previously employed) and 1 (previously unemployed).

## Results

Table 1 presents the descriptive statistics and correlations between the study variables. Among the demographic variables, age is negatively related to proactive coping (*r*s between *r* = -.29 and *r* = -.32), and negatively related to unmet expectations, *r* = .22, *p* = .005 (Time 1), respectively, *r* = .22, *p* = .018 (Time 2). The high correlations of proactive coping between the assessments indicate the stability of this construct over time (*rs* between *r* = .71 and *r* = .85), whereas the measures of unmet expectations are interrelated to a lesser extent between the assessments (*r*s between *r* = .08 and *r* = .41). Table 1 revealed a negative association of proactive coping and unmet expectations, especially at Time 1, *r* = -.33, *p* < .001, and Time 2, *r* = -.32, *p* < .001.

**Table 1:** Means (M), standard deviations (SD), and correlations between study variables.

Variable	*M*	*SD*	1	2	3	4	5	6	7	8
1. Gender^a^	0.53	0.50								
2. Age	31.47	9.79	-.08							
3. Pre-entry experience^b^	0.44	0.50	-.07	.22**						
4. Proactive coping (t1)	2.88	0.45	-.10	-.31**	-.11					
5. Proactive coping (t2)	2.94	0.50	.11	-.29**	-.18	.71**				
6. Proactive coping (t3)	2.88	0.52	.20*	-.32**	-.19	.72**	.85**			
7. Unmet expectations (t1)	-0.05	1.79	-.03	.22**	-.01	-.33**	-.46**	-.35**		
8. Unmet expectations (t2)	-0.08	2.73	.06	.22*	.00	-.31**	-.32**	-.30**	.41**	
9. Unmet expectations (t3)	0.80	3.92	.09	.05	-.11	-.07	-.01	-.01	.08	.31**

*Notes*.

^a^ Gender is dummy-coded (1 = male, 0 = female)

^b^ pre-entry experience is dummy-coded (1 = previously unemployed, 0 = previously employed)

* *p* < .05

** *p* < .01.

### Change in unmet expectations

The participants received questionnaires at three times. Accordingly, we computed multilevel models for longitudinal data, with the respective waves of assessment on Level 1 and the subjects on Level 2 [31]. We entered the control variables, namely gender, age, and pre-entry experience at Level 2. Proactive coping as a timely varying covariate was included on Level 1 in the analyses. In order to control for changes over time, we considered the linear and quadratic effect of time.

Table 2 shows the results of multilevel models predicting the change of unmet expectations over time. In Model 1a we controlled for gender, age, and pre-entry experience. Among those variables, age is positively related to unmet expectations, *b* = 0.05, *p* < .001, whereas pre-entry experience (i.e. being unemployed before entering the organization) has no effect on unmet expectations, *b* = -0.30, *p* = .293. In Model 1b we included the linear and quadratic effects of time. The linear effect of time was not significant, which indicates no linear change of unmet expectations over time, *b* = -0.57, *p* = .245. Thus, Hypothesis 1 was not confirmed. However, the positive quadratic effect of time is significant and outlines a timely limited decrease in unmet expectation at Time 2, *b* = 0.48, *p* = .046.

**Table 2:** Effects of time and proactive coping on unmet expectations.

Predictor	Model 1a	Model 1b	Model 1c	Model 1d
Level 1 (within-subjects)				
(Intercept)	1.43**	1.43**	2.74*	2.58*
Time		-0.57	-0.38	4.37
Time x Time		0.48*	0.40	-2.93*
Proactive coping			-1.25**	-1.19**
Time x Proactive coping				-1.63
Time x Time x Proactive coping				1.15*
Level 2 (between-subjects)				
Gender^a^	0.10	0.08	0.04	0.03
Age	0.05**	0.05**	0.03*	0.03*
Pre-entry experience^*b*^	-0.30	-0.31	-0.38	-0.38
Deviance (-2 log-likelihood)	1780.18	1773.30	1755.54	1747.51
Change in Deviance		6.88*	17.76**	8.03*
*AIC*	1796.18	1793.30	1777.54	1773.51
*N*	380	380	380	380

*Notes*. Only fixed effects are reported; coefficients are unstandardized

^a^Gender is dummy-coded (1 = male, 0 = female)

^b^pre-entry experience is dummy-coded (1 = previously unemployed, 0 = previously employed)

* *p* < .05

** *p* < .01.

### Unmet expectations and proactive coping

Hypothesis 2 assumes that proactive coping is negatively related to unmet expectations. In Model 1c we included proactive coping as an additional predictor, which revealed the negative influence of proactive coping on unmet expectations, *b* = -1.25, *p* < .001 (cf. Table 2). Thus, Hypothesis 2 was confirmed; there is no evidence for a general relation between proactive coping and unmet expectations.

**Fig 1:**
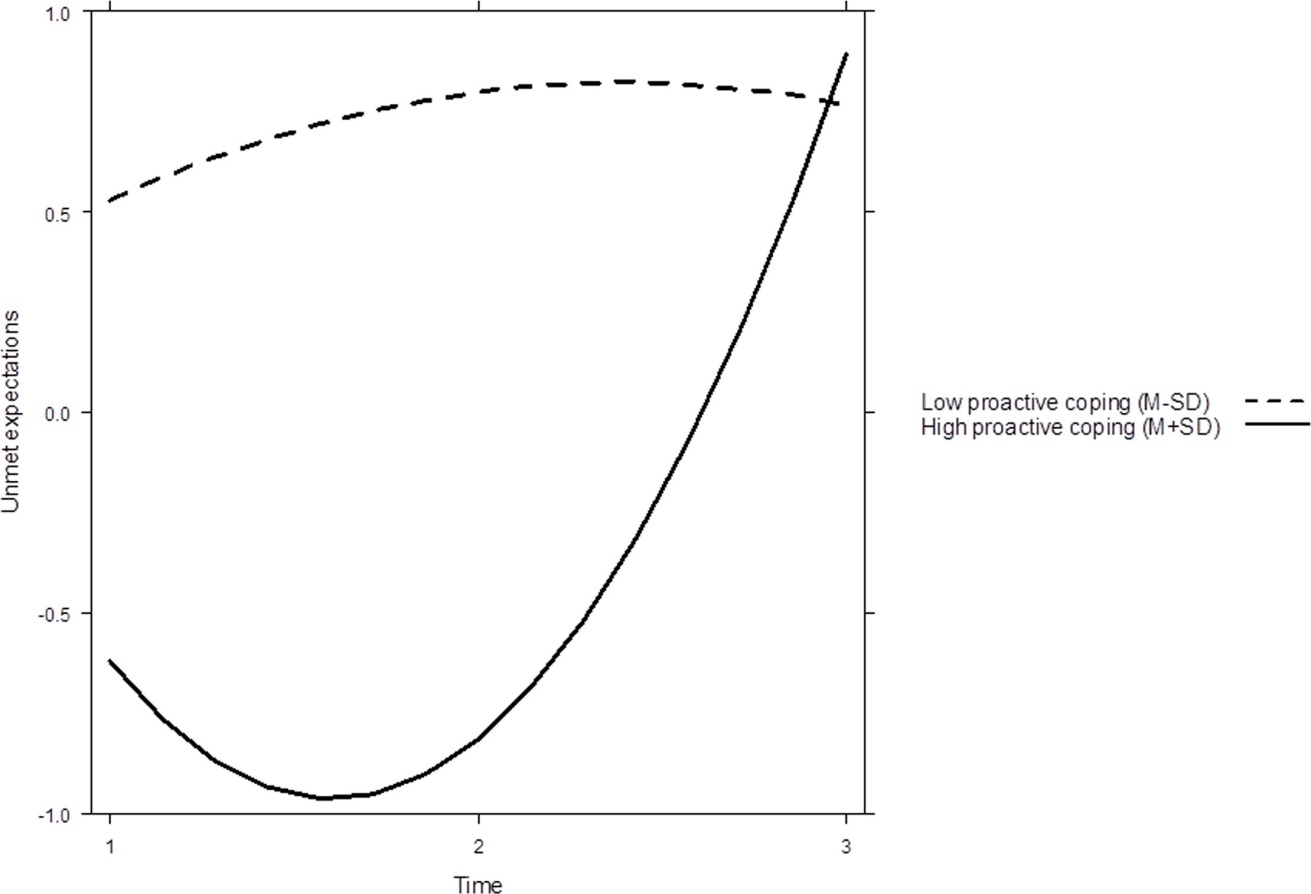
Change of unmet expectations over time in dependence of proactive coping.

Hypothesis 3 assumed, that proactive coping is related to a lower increase in unmet expectations. To gain further insight into the change on unmet expectations in dependence of proactive coping, we additionally considered the interaction of proactive coping with the linear and quadratic effects of time. Model 1d revealed no interaction between the linear effect of time and proactive coping, *b* = -1.63, *p* = .093 (cf. Table 2). Thus, Hypothesis 3 was not supported; a higher level of proactive coping is not related to a lower increase in unmet expectations. However, Model 1d revealed an interaction effect between the quadratic effect of time and proactive coping, *b* = 1.15, *p* = .014. Since this effect is positive, unmet expectations evolved differently in time especially for participants high in proactive coping. Fig 1 depicts the predicted change of unmet expectations for individuals low in proactive coping (*M-SD*) compared to individuals high in proactive coping (*M+SD*). Individuals with a low level of proactive coping reported generally higher levels of unmet expectations. Most interestingly, in the case of high proactive coping, unmet expectations slightly decrease between Time 1 and Time 2, but in the long run increase to a level comparable to individuals with low proactive coping.

## Discussion

Unmet expectations play a significant role in influencing organizational behavior [3, 13, 18]. Traditionally, the level of fulfillment of newcomers’ initial expectations has been measured before and just after organizational entry [32]. Since matching one’s own expectations with organizational reality might depend on dispositional factors as well as on the situational context, the discrepancy between job previews and encountered working conditions could change in time. Especially during the entrance phase, newcomers are part of onboarding programs that provide support and ensure the fulfillment of their expectations. However, as these onboarding programs end, newcomers are confronted with the actual working conditions and thus the reference for the assessment of their expectations changes.

In the present study, we considered the fulfillment of newcomers’ expectations as a process and assessed unmet expectations three times within the first six months of a new employment. Our results outlined a change in the fulfillment of employees’ expectations over time. More precisely, after an initially low level of unmet expectations, new employees indicated a higher level after six months in the new workplace. This change in unmet expectations might be explained by the stage model described by Korte, Brunhaver and Sheppard [33]. At the pre-entry stage, potential recruits anticipate future job characteristics and build expectations about what they will experience. The subsequent entry stage involves the confrontation of prior expectations with reality, and finally, the integration stage includes making sense of gathered information and adopting to new roles. As it has been mentioned before, when new employees step into the organization they are likely to be more satisfied and self-efficient because of the mere experience of being hired. This entrance phase could be compared to a honeymoon phase [14, 34]. In this phase, dissatisfying aspects of the new job are likely to be less recognized and the attendance of job orientation programs might create an impression of a supportive work environment. However, after getting more involved into the new job, organizational entrants experience the actual working conditions and social interactions are focused on task fulfillment, which could increase the difference between initial and current expectations. Accordingly, research points out a honeymoon-hangover effect [34], which could find its expression also in an increased level of unmet expectations.

Our results outlined an influence of proactive coping on unmet expectations. In particular, proactive coping was associated a higher fulfilment of expectations, which was reflected in an initially lower level of unmet expectations. Furthermore, our results revealed proactive coping as an influential factor of the development of unmet expectations. Persons with a low level of proactive coping indicated a higher level of unmet expectations at early stages of organizational entrance. Contrary, persons high in proactive coping reported a lower level of unmet expectations after organizational entry, which increased again at later stages. As already mentioned, in the entry phase onboarding programs support and appreciate newcomers’ initiative. However, the termination of these programs and the experience of the real working conditions could lead to disappointment, which might be especially true for persons high in proactive coping whose proactive behaviors are cut down in their effectiveness. To put it more simply, unmet expectations increase because the organization does not respond to proactive behaviors any more.

### Limitations

Several potential limitations of the present research should be kept in mind. First, data were obtained from a relatively small sample. However, we reached all organizational newcomers of the company who were hired in newly opened stores in the entire country. Second, we relied on self-reports for assessing both dependent and independent variables. Self-reports tend to have some disadvantages that were described in the literature with scrutiny (*e*.*g*., dependence to introspective abilities of respondents, susceptibility to response styles and faking answers, especially in a socially approved way). However, the cases mentioned above seem to be rarely a serious problem in most research settings [35] and may be overcome by verification of psychometric properties of the instruments [36]. Nonetheless, the self-report measures are often the only possible way to examine psychological constructs such as self-efficacy or own expectations in a specific context. Expectations are traditionally measured by using self-report methods including direct questions about individuals’ thoughts and previews about future job. In the case of the presented study, all measures have well-assessed psychometric properties (in terms of reliability and validity) and were previously used in other research settings in which job search behavior was studied. Since we were primarily interested in newcomers’ perceptions and subjective evaluations of their employment situation, the use of self-report data is reasonable. Next, in our study we focused on the role of proactive coping on unmet expectations. We assumed that proactive copers scan the environment to detect important and challenging difficulties, and engage in own efforts or skills to deal with those difficulties [8]. Therefore, we expected proactive coping having an influence on unmet expectations. However, an opposite direction could be plausible. In particular, unmet expectations can refer to a lower job satisfaction and higher job stress, and thus may influence proactive coping strategies [7]. Unsatisfied organizational newcomers might therefore engage in proactive behaviors to change their own situation. In order to address these both directions of effects, we additionally computed a cross-lagged panel analysis. The results indicated that both directions are present but only between waves 1 and 2. As predicted, proactive coping at Time 1 had an influence on unmet expectations at Time 2, *B* = -1.37, *p* = .023, while controlling for the influence of unmet expectations at Time 1. However, unmet expectations at Time 1 had an influence on proactive coping at Time 2, *B* = -0.06, *p* = .006. Thus, both directions seem possible. In order to scrutinize the direction of effects between proactive coping and unmet expectations, future research could conduct more sophisticated analyses such as a bivariate latent change score model [37].

Finally, we had no information on the work environment and socialization tactics. However, there should be no difference in working environments because the participants were hired for the same positions within the same company and the work environment was standardized, and thus, comparable throughout the whole organization.

### Implications

Our findings have several implications. Unmet expectations can negatively influence newcomers’ outcomes, like job satisfaction and turnover intention. Neff and Geers [17] reviewed that highly optimistic expectations can sometimes lead to a false sense of security. More specifically, participating in onboarding programs can prevent newcomers form perceiving and tackling difficulties in the workplace, and therefore, cause a context in which problems are left unresolved, and thus increase over time. Given the importance of the fulfilment of newcomers’ expectations, existing theory should be broadened to account for the change of unmet expectations. From a practical perspective, organizations are supposed to keep newcomers’ expectations on a realistic level. Also, research on organizational socialization suggests that organizations should lower expectations of potential employees so that they are more likely to be met [13]. In this vein, realistic job previews are an effective means to lower initial expectations [38]. Another starting point might be the improvement of newcomers’ proactive coping, which is related to a lower level of unmet expectations, particularly at early stages of the onboarding process. Proactive coping could be enhanced by intervention programs [21]. However, the influence of proactive coping on unmet expectations is limited to an early stage of organizational onboarding. From an interactionist perspective between personal and situational factors [29], proactive coping should be appreciated and supported by the organization, in order to prevent a honeymoon-hangover effect in the long run.

## Conclusion

In the present research we sought to understand the change of unmet expectations of organizational newcomers. Though proactive coping is an effective means to prevent new employees from a high level of unmet expectations, the sweet honeymoon at early stages of organizational entry might be followed by a hangover effect in the long run. Proactive coping is a crucial factor in this process, which should be appreciated as a valuable dowry and consolidated in the organization.
